# Relationship between sex differences in drinking, smoking, and exercising and the incidence of malignancies and medical procedures: a cross-sectional study of 21,916 participants in China

**DOI:** 10.7150/jca.95456

**Published:** 2024-06-17

**Authors:** Mingyan Hao, Yifan Li, Wenjun Ma, Lizheng Wang, Janzhong Zheng, Yibo Wu

**Affiliations:** 1 School of Public Health, Shanxi Medical University, Shanxi Hospital Affiliated to Carcinoma Hospital, Chinese Academy of Medical Sciences, Shanxi Province Carcinoma Hospital, Carcinoma Hospital Affiliated to Shanxi Medical University, Taiyuan, 030001, China.; 2 Hepatobiliary, Pancreatic and Gastrointestinal Surgery, Shanxi Hospital Affiliated to Carcinoma Hospital, Chinese Academy of Medical Sciences, Shanxi Province Carcinoma Hospital, Carcinoma Hospital Affiliated to Shanxi Medical University, Taiyuan, Shanxi, 030013, China.; 3 School of Public Health and Preventive Medicine, Shanxi Medical University, Taiyuan, 030001, China.; 4 Shanxi Hospital Affiliated to Carcinoma Hospital, Chinese Academy of Medical Sciences, Shanxi Province Carcinoma Hospital, Carcinoma Hospital Affiliated to Shanxi Medical University, Taiyuan, Shanxi, 030013, China.; 5 School of Public Health, Peking University, Beijing 100191, China.

**Keywords:** malignancy, sex difference, tea consumption, coffee consumption, sugary beverages

## Abstract

**Objectives:** The unresolved issue of the relationship between sex differences in tea, coffee, and beverage consumption and malignancy risk prompted our study in 2022.

**Methods:** Logistic proportional hazards models were used to estimate odds ratios (ORs) and 95% confidence intervals (CIs) in our investigation of the associations between cancer risk and tea, coffee, and beverage consumption.

**Results:** Our findings revealed that frequent consumption of white tea significantly reduced the occurrence of malignant tumours, but this effect was detected only in the fully adjusted model for males (OR: 0.736, 95% CI: 0.095-5.704). The amount of sugar added to coffee was associated with an increased risk of malignancy in a dose-dependent manner (P for trend = 0.001), with significance observed for both men (P for trend = 0.049) and women (P for trend = 0.005) in the final model. Notably, individuals who consumed more than 2100 ml of sugary beverages daily had a statistically significant reduction in malignancy risk (OR: 0.219, 95% CI: 0.052-0.917). Interestingly, the intake of sugary beverages had a protective effect on cancer incidence, with a significant effect on males (P for trend = 0.031) but not females (P for trend = 0.096) in the final model.

**Conclusions:** Our study highlights the substantial impact of regular white tea consumption on reducing the risk of malignant tumours in males. This study first reported that the potential protective effect of consuming sugary beverages is predominantly observed in males, and a correlation between the amount of sugar added to coffee and a heightened risk of malignancy.

## Introduction

According to the 2023 Chinese National Cancer Report 1, both the number of cancer cases and deaths in China are continuing to increase. The annual medical expenditure caused by cancer has now exceeded 220 billion yuan. These trends can be attributed to socioeconomic development, changes in lifestyle, and the aging population.

Among lifestyle factors, drinking habits, including tea, water, coffee, and other beverages, play a significant role 2. Numerous meta-analyses of epidemiological studies have been conducted in recent years to examine the relationship between tea consumption and cancer outcomes 3,4,5. Accumulating evidence from cellular, animal, clinical, and epidemiological research suggests that tea consumption is associated with various health benefits 6,7,8,9. These benefits include chemoprevention of cancers 10, a reduction in chronic inflammation 11, protection against heart and liver diseases 12, management of diabetes, prevention of neurodegenerative diseases, protection against skin aging induced by UVB radiation, and prevention of bone fracture 13. Furthermore, tea consumption has been found to have other beneficial effects, such as chemosensitizing, antioxidant, and stress-reducing effects 14.

Coffee and tea are popular drinks that are consumed globally 15. A breast cancer diagnosis revealed a link between increased coffee consumption and enhanced breast cancer and overall survival rates 16. Likewise, greater tea consumption post-diagnosis may be linked to improved overall survival. Nevertheless, the study delves into sex disparities in the connection between drinking habits and cancer occurrence. In essence, drinking behaviours can be both good and bad for Chinese residents. The ultimate aim of our research was to determine whether different drinking behaviours of males and females are associated with the incidence of cancer.

## Methods

### Study population

The study was performed on individuals from the Chinese psychological and behavioural survey, which included 10,958 males and 10,958 females in its population-based cohort. The study spanned from July 10, 2022, to September 15, 2022, and included individuals from all 22 provinces, 5 autonomous regions, and 4 municipalities across mainland China. To execute the research, survey teams, consisting of no more than 10 members, were openly recruited and rigorously trained within the designated sample cities. In each city, at least one investigator or survey team was enlisted, whereby the former collected 30-90 questionnaires and the latter collected 100-200 questionnaires in their respective areas.

We employed Wenjuanxing, the survey platform in China (https://www.wjx.cn/ accessed on July 1, 2022). to conduct our survey. Our team personally distributed the questionnaires to residents, carefully maintaining adherence to ethical practices. Each participant was required to sign a consent form to ensure that the participants provided informed consent. The questionnaire was made available online through a provided link, and we diligently recorded the unique questionnaire number for every respondent. In situations where participants had the cognitive ability to comprehend the questions but could not respond, we conducted one-on-one interviews and captured their answers on their behalf.

**Figure 1:**
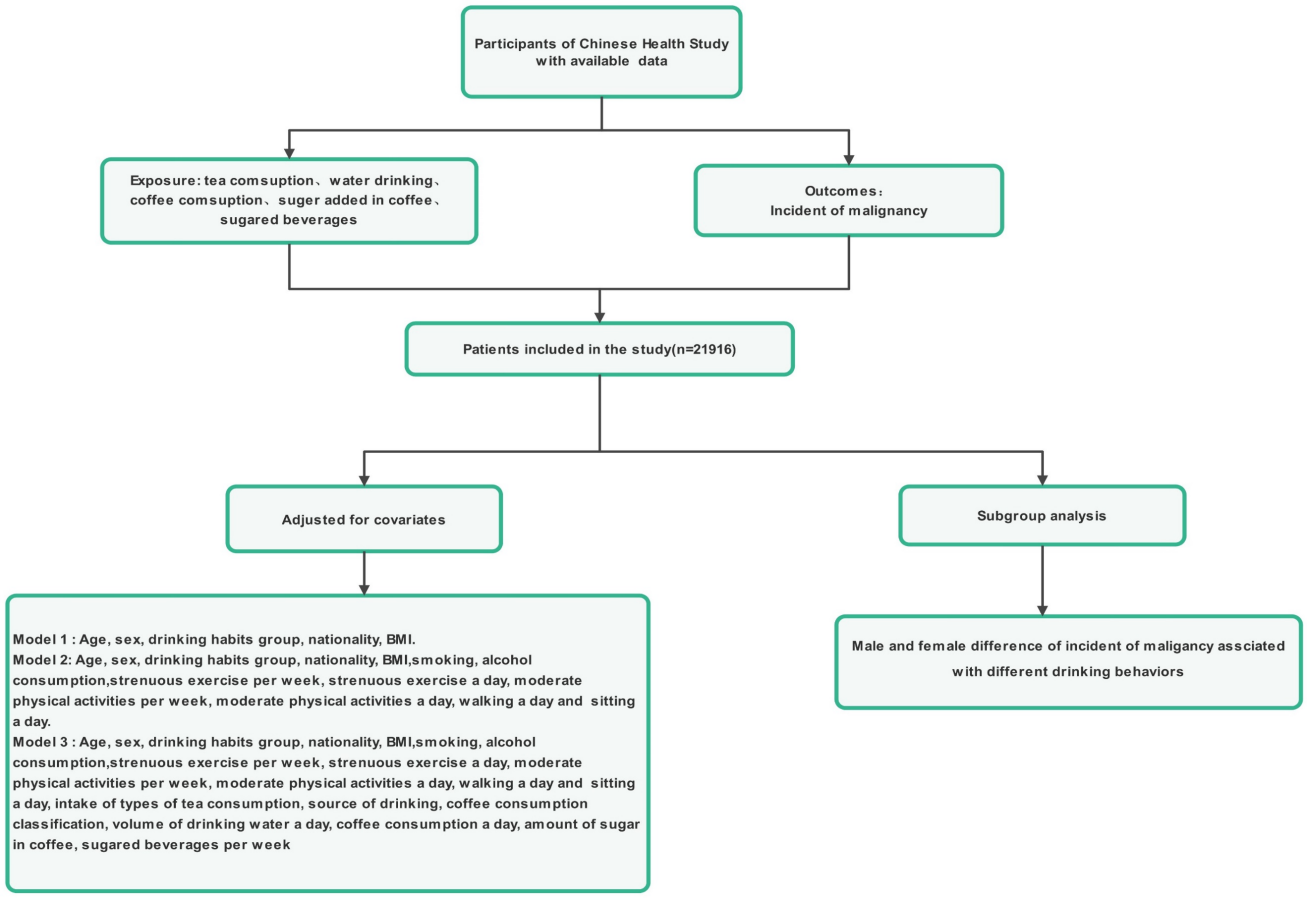
Flowchart for the enrolment of participants

The inclusion criteria for participation in the study were people aged more than 12 years old, permanent residents, and who travelled for ≤1 month annually. Participation in the study was voluntary, as the participants had the freedom to either independently complete the online questionnaire survey or seek assistance from the investigators if required and were capable of understanding the importance of each question within the survey (Figure 1). The reasoning behind selecting teenagers aged 12 years and older for our study is outlined below. First, we have observed that both teenagers and adults now embrace death education, benefiting from an improved-quality education and an overall societal environment that encourages open conversations about this topic. Teenagers aged 12 years and older have the means and environment necessary to develop their perspectives on life and death, which could lead to mature ideas about hospice care. Second, these teenagers can understand and independently respond to the questionnaire, particularly if they have access to a smartphone. The exclusion criteria were individuals who (1) suffer from mental illnesses or have insanity, (2) are enrolled in analogous research projects, or (3) refuse to collaborate.

### Statistical analysis

Data analysis for this research utilized SPSS™ software, specifically version 25.0, by SPSS, Inc., Chicago, IL, USA. Statistical analysis involved using descriptive statistics to calculate the mean values and standard deviations (SDs) of continuous variables and determining p values for each variable. The comparative analysis was performed using ANOVA and t-tests to assess the relationships between factors and malignancy-related drinking behaviours. Logistic regression was performed to investigate variables connected to malignancy occurrence, with entry criteria set at P = 0.05 and removal criteria set at P = 0.01.

## Results

In this study, a total of 21916 participants from the primary care dataset were included at baseline (Figure 1). The distribution ratio of men to women was 1:1, 9.5% of participants were 12-17 years old, 71.4% of participants were 18-59 years old, and 19.2% of participants were above 60 years old. Table 1 displays the baseline features of participants categorized by the presence of cancer. Participants who had malignancies were older, more likely to smoke, more likely to consume tea, more likely to drink water, more likely to consume coffee, added a greater volume of sugar to coffee, and more likely to drink sugary beverages. Malignancy is also associated with higher BMIs, more frequent use and weekly use, less walking, and more sitting. No notable differences in sex, age, or nationality distribution were observed among participants with normal health status and those with malignancies.

**Figure 2:**
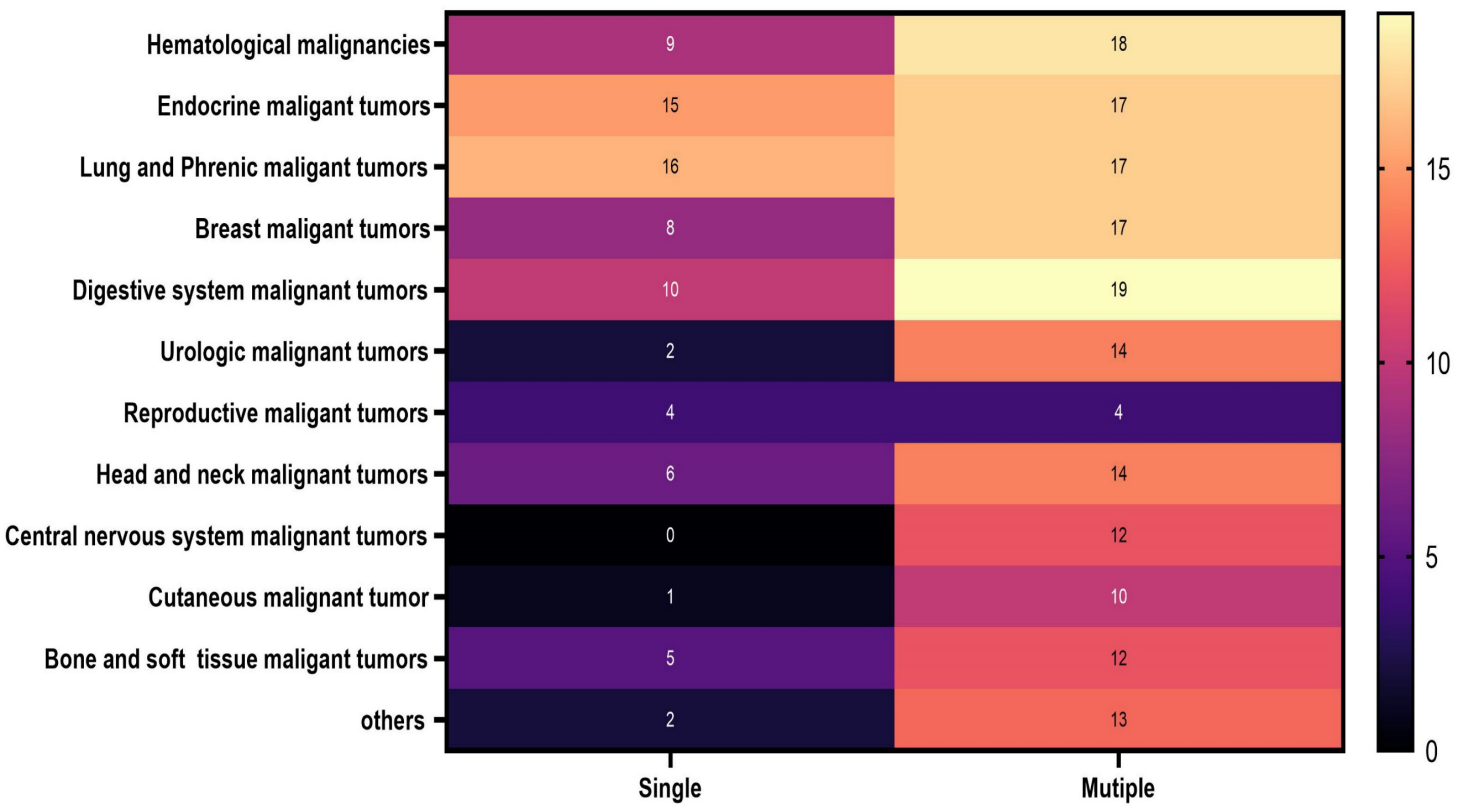
The distribution of all participants in 2022

**Figure 3:**
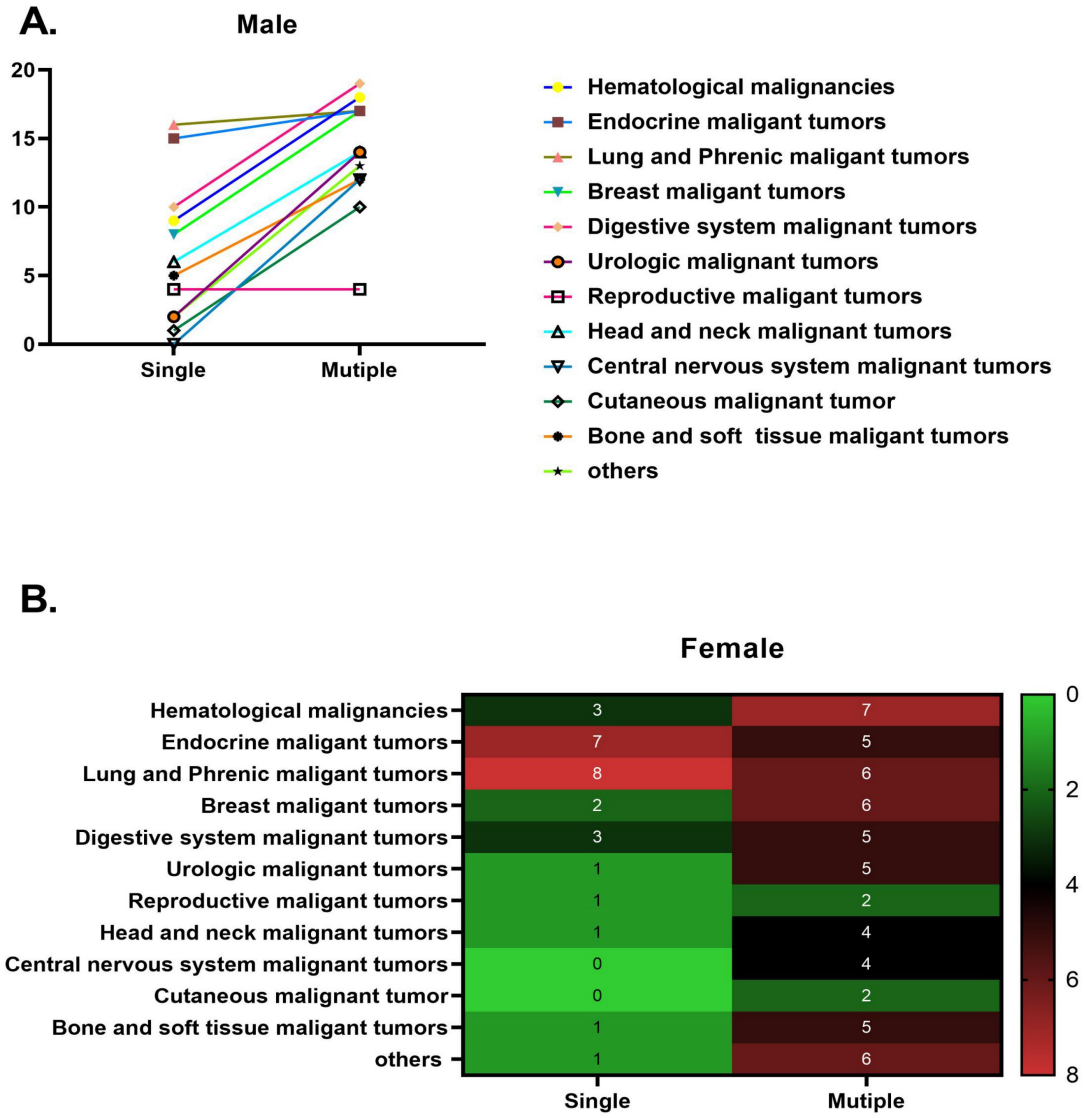
The distribution of malignancies among male and female participants in 2022: A. male and B. female

Specific information on the malignant cancers included in this study is clearly shown in Figure 2. Details regarding the malignant cancers of men and women examined in this study are prominently displayed in Figure 3.

Table 2 presents the findings of our study examining the relationship between the incidence of cancer and the consumption of tea, coffee, and other beverages. Specifically, we analyzed the impact of the addition of sugar to coffee on the risk of malignancy and observed a dose-dependent association (P for trend = 0.001). Our results indicated that individuals who regularly added more sugar to their coffee face a significantly elevated risk of developing malignancies. Notably, this risk increased with the frequency of coffee consumption and the quantity of sugar added per day. For instance, when comparing individuals who added more than 5 g of sugar to their coffee (including subgroups with 5 g, 10 g, and 15 g of sugar added), we observed a significantly greater risk of malignancy among daily coffee drinkers who added larger volumes of sugar. These findings suggest that excessive consumption of sugar in coffee may contribute to an increased risk of cancer. Therefore, limiting the amount of sugar added to coffee and considering potential alternatives are advisable to reduce this risk. For the 15 g/day group with the highest sugar intake, the odds ratio (OR) was 9.362 (95% CI: 2.680-32.709). Compared with those who did not consume sugary beverages, daily consumers of more than 2100 ml of sugary beverages had a significantly reduced risk of malignancy (OR: 0.219, 95% CI: 0.052-0.917). We observed that the risk estimates for individuals who consumed less than 900 ml of sugary beverages daily (OR: 0.553, 95% CI: 0.366-0.835) and those who consumed between 900-1800 ml (OR: 0.365, 95% CI: 0.164-0.812) were similar. Moreover, the risk of cancer increased as the consumption of sugary beverages and the amount of sugar added to coffee increased. Similarly, the amount of water consumed was closely linked to a greater incidence of cancer (P for trend=0.027), especially for those who drank more than 2100 ml of water (OR:2.253, 95% CI: 1.130-4.493, P=0.021), than for those who drank less water (≤1200 ml). However, no notable variation in the likelihood of cancer was observed for participants consuming 1200-1499 ml (P=0.878), 1500-1699 ml (P=0.210), or 1700-2099 ml (P=0.492) of water. The consumption of yellow tea (OR: 4.033, 95% CI: 1.102-14.765, P=0.035) and compressed tea (OR: 2.023, 95% CI: 1.250-3.273, P=0.004) was significantly correlated with an increased risk of cancer.

**Figure 4:**
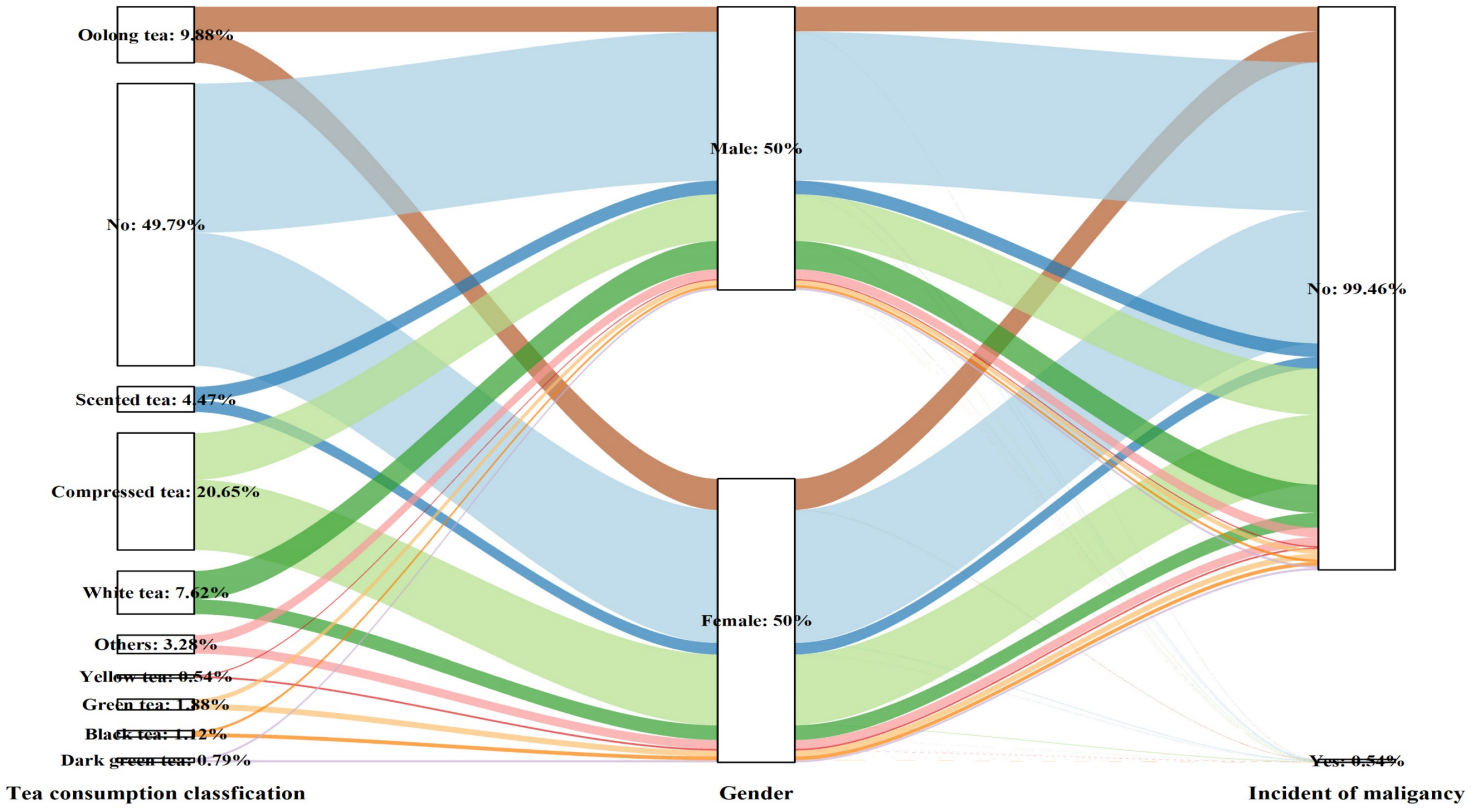
Sankey plot of sex differences in tea consumption classification and the risk of malignancy

Various habits of tea consumption had varying effects on the incidence of malignancy based on sex differences (Table 3 and Figure 4). Among females, except for those who chose secreted tea (OR: 2.909, 95% CI: 1.028-8.232, P=0.044), different tea consumption choices did not seem to have any negative effects on the incidence of malignancy compared to non-tea drinkers. However, in males, different tea consumption patterns were strongly linked to greater chances of developing cancer—malignancy (p for trend= 0.033). Specifically, the groups consuming black tea (OR: 5.563, 95% CI: 1.479-20.925, P=0.011), yellow tea (OR: 7.350, 95% CI: 1.739-31.063, P=0.007), dark green tea (OR: 5.453, 95% CI: 1.04-27.192, P=0.039), and compressed tea (OR: 2.680, 95% CI: 1.358-5.286, P=0.004) had the highest risk of malignancy in the fully adjusted model (Model 3).

**Figure 5:**
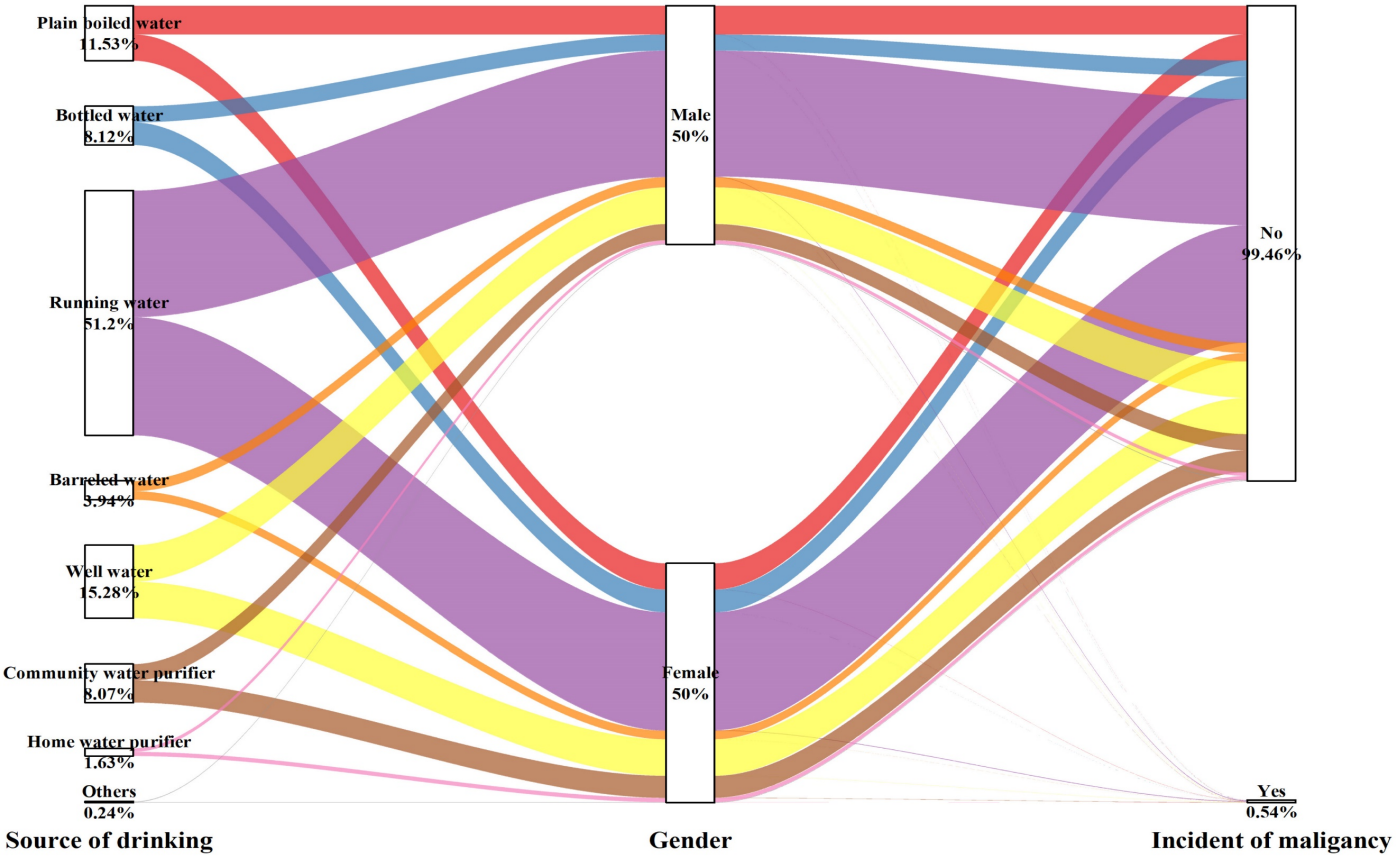
Sankey plot of sex differences in the source of drinking water and risk of malignancy

Table 4 and Figure 5 show that the choice of the drinking water source had no impact on the occurrence of malignancy for either men (P for trend= 0.201) or women (P for trend= 0.949), except for the male subgroup using community water purifiers, which showed significant outcomes (OR: 2.448, 95% CI: 1.179-5.081, P=0.016).

**Figure 6:**
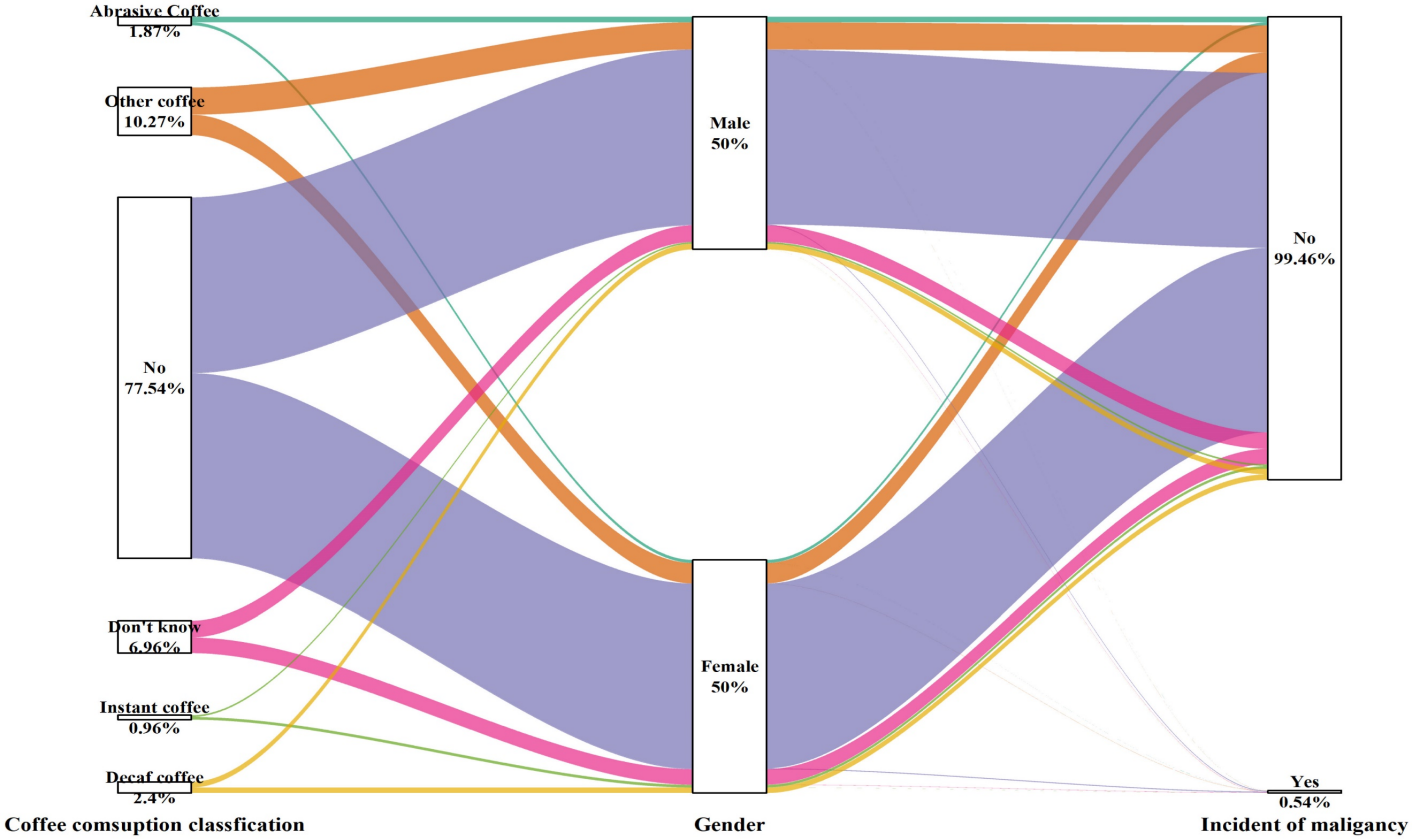
Sankey plot of sex differences in the coffee consumption classification and risk of malignancy

The type and amount of coffee consumed were not related to the development of cancer in either male or female participants (Table 5 and Figure 6).

**Figure 7:**
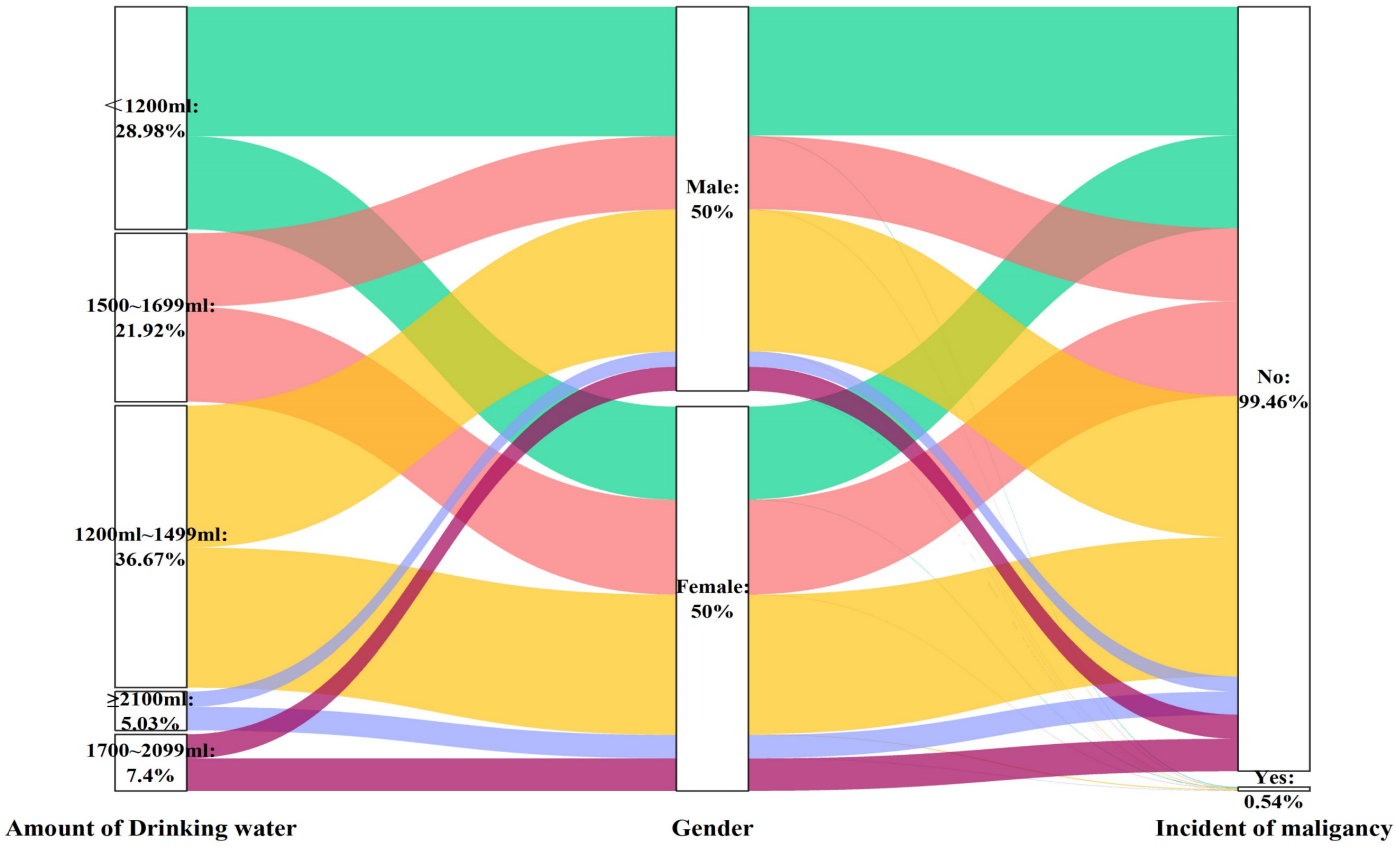
Sankey plot of sex differences in the volume of drinking water and risk of malignancy

Similarly, the volume of water consumed was not associated with the likelihood of developing cancer in individuals of either sex (Table 6 and Figure 7), except for the male subgroup who consumed more than 2100 ml of water (OR: 2.989, 95% CI: 1.150-7.770, P=0.025), in the fully adjusted model (Model 3).

**Figure 8:**
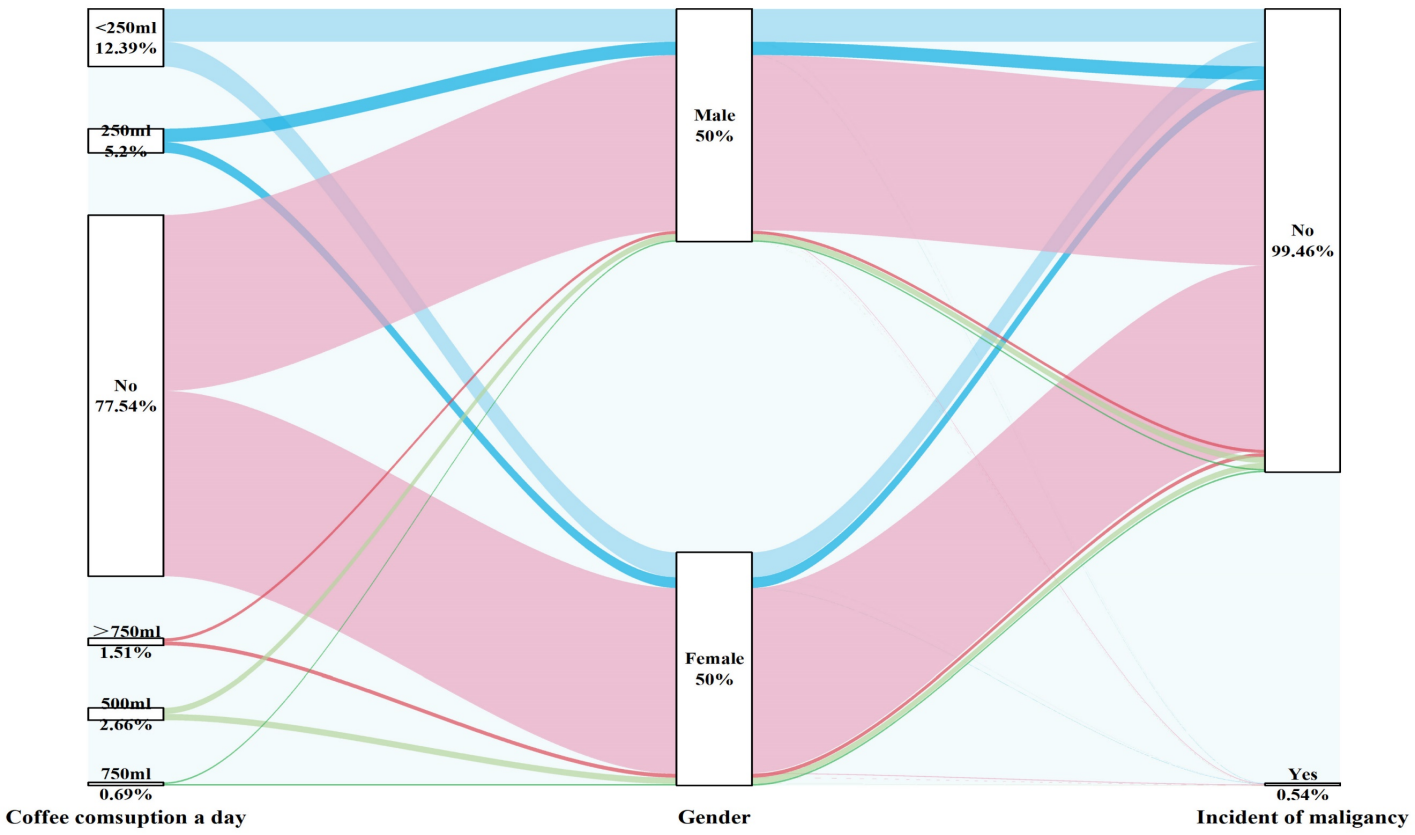
Sankey plot of sex differences in daily coffee consumption and the risk of malignancy

The amount of coffee consumed daily was not related to the incidence of malignancy, regardless of sex, and this effect remained consistent in both the crude and fully adjusted models (Figure 8).

**Figure 9:**
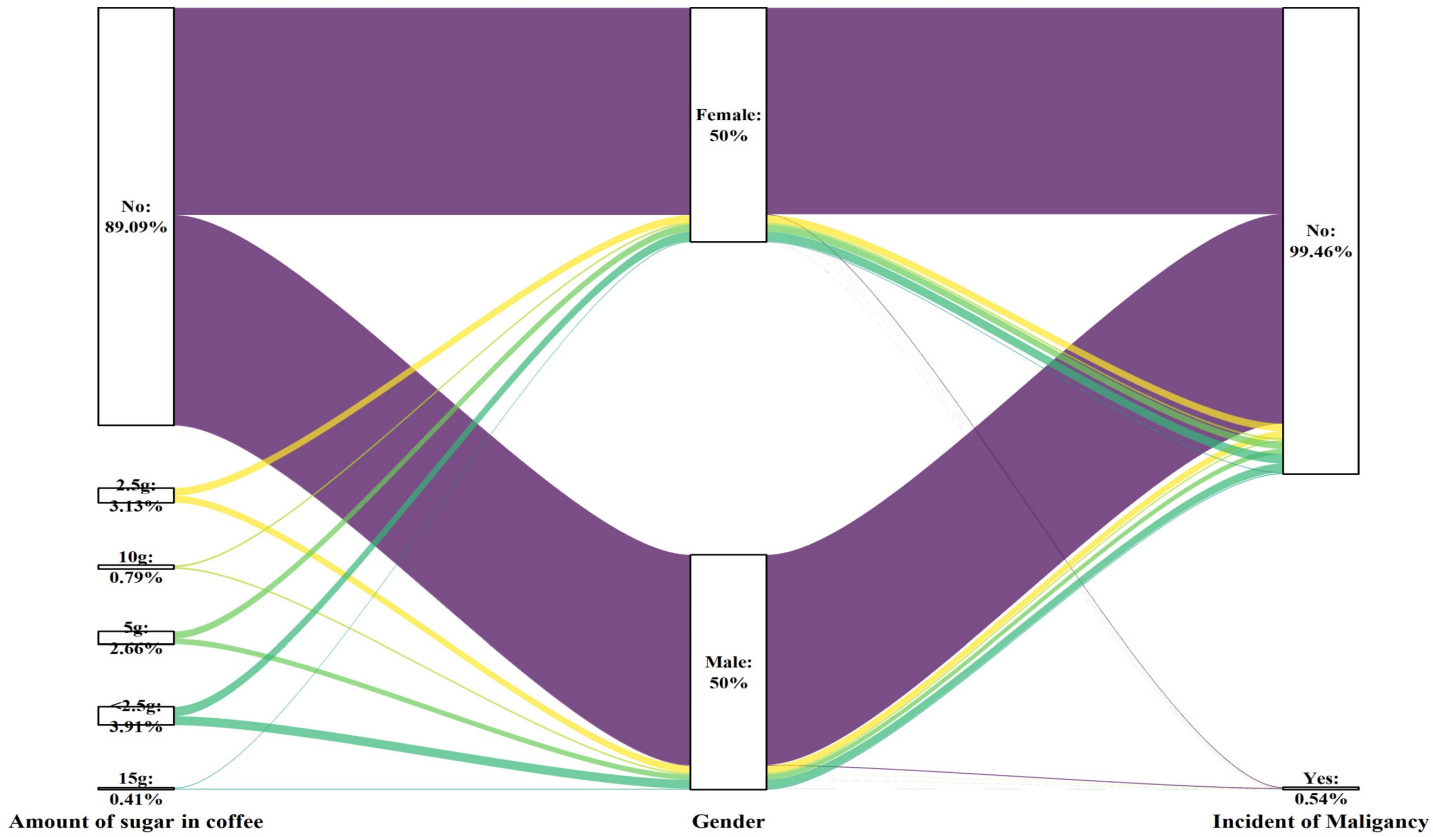
Sankey plot of sex differences in the amount of sugar added to the coffee and the risk of malignancy

A notable correlation between cancer incidence and the level of sugar added to coffee was observed among both genders (p for trend= 0.049 for males, p for trend= 0.005 for females). However, after adjusting for various factors (Model 3), the biological effects varied among the different groups, depending on the amount of sugar added to the coffee. In the male subgroup, consuming 15 g of sugar (equivalent to three teaspoons) added to coffee had potentially harmful effects on the germination of malignancy (P=0.003). In the female population, the consumption of 10 g of sugar (equivalent to two teaspoons) in coffee was correlated with a heightened cancer risk (P<0.001). This information is presented in Table 7, as well as Figures 9 and 11.

**Figure 10:**
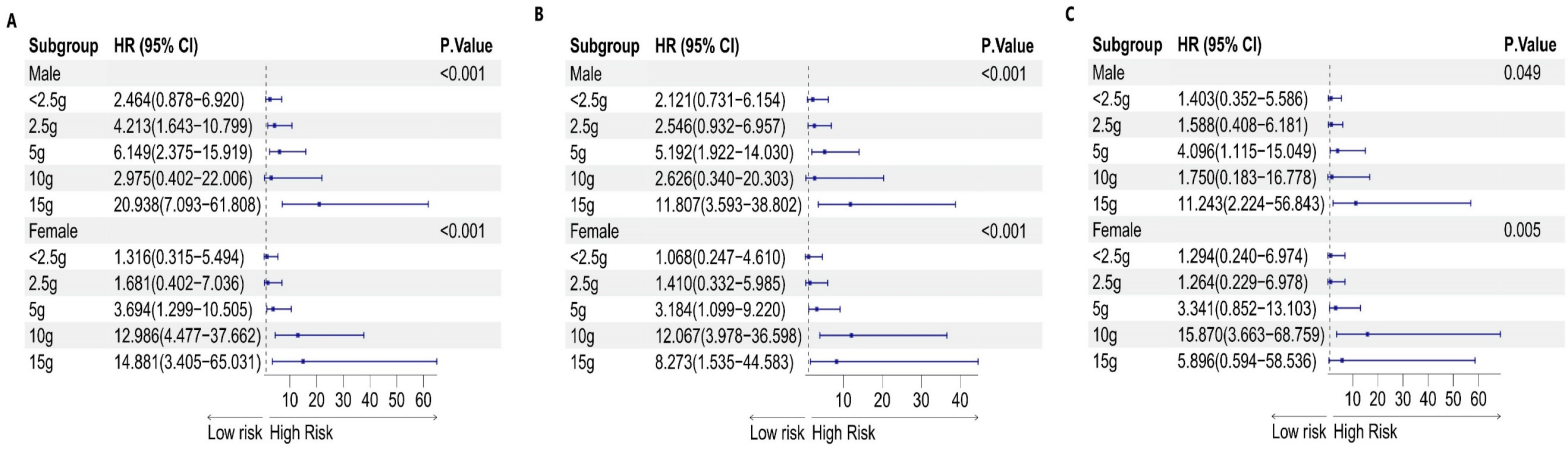
Sankey plot of sex differences in the consumption of sugary beverages per week and the risk of malignancy

**Figure 11:**
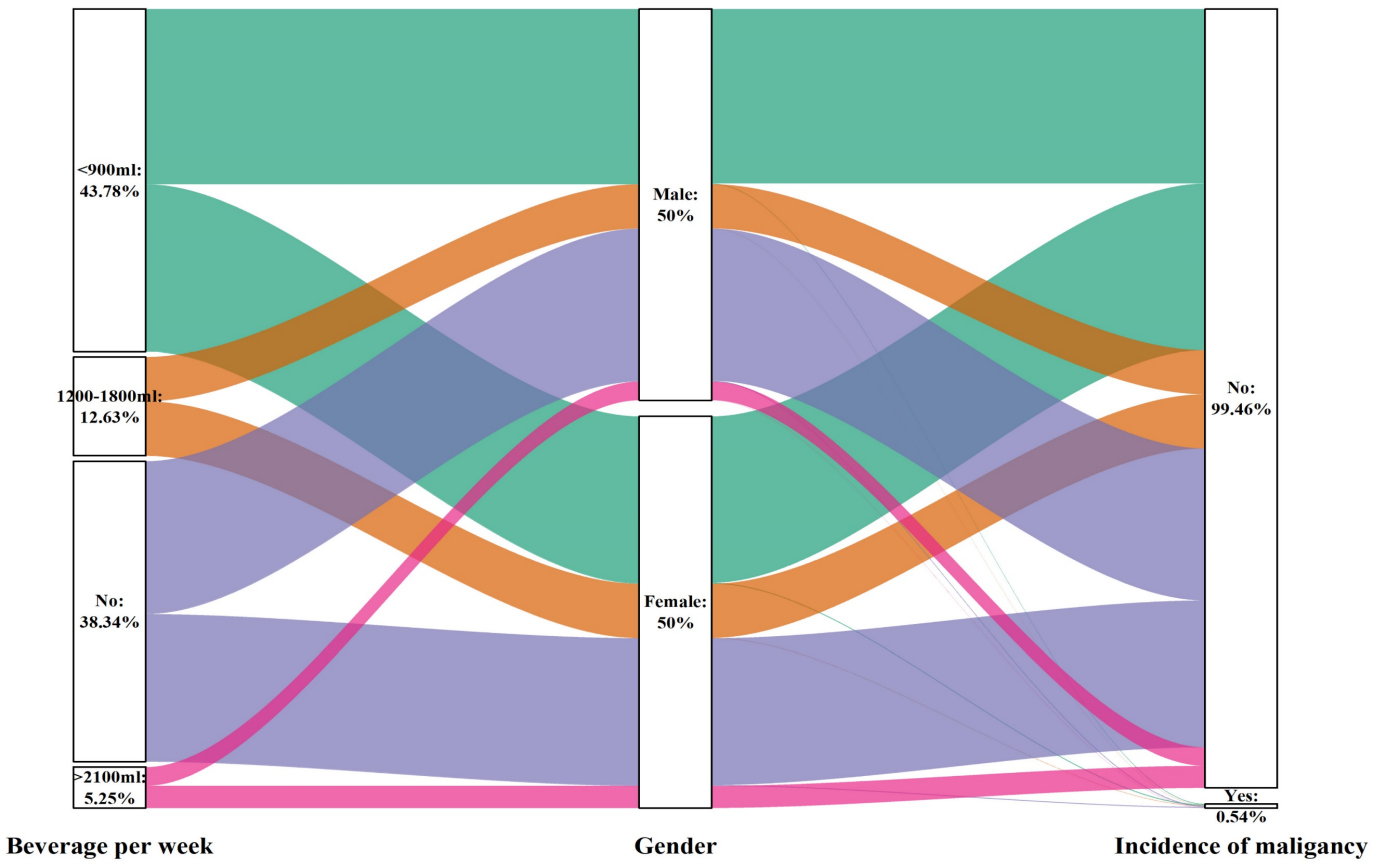
Forest plot of sex differences in the amount of sugar added to coffee and the risk of malignancy: A. Model 1, B. Model 2, and C. Model 3

**Figure 12:**
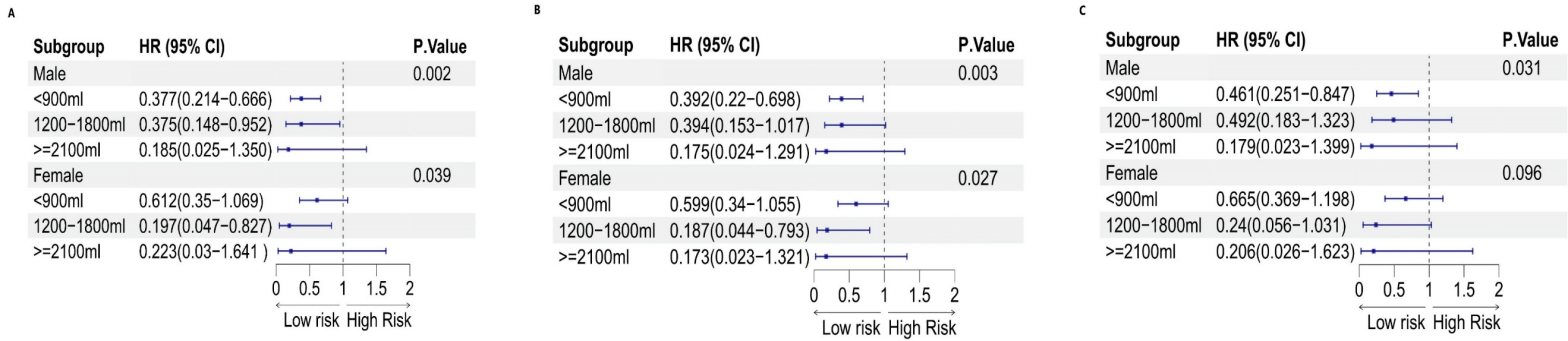
Forest plot of sex differences in sugary beverage consumption per week and the risk of malignancy: A. Model 1, B. Model 2, and C. Model 3

On the other hand, the intake of sugary beverages may have a protective effect on the occurrence of cancer. This effect was significant for men (P for trend= 0.031) but not for women (P for trend= 0.096). For men who consumed less than 900 ml of sugary beverages, a 54% reduction in the chance of developing cancer was observed (OR: 0.461, 95% CI: 0.251-0.847; P=0.013). This information is presented in Table 8, as well as Figures 10 and 12.

## Discussion

Tea, a widely consumed traditional beverage, possesses a wide array of health benefits due to its natural attributes 17. Research has primarily concentrated on the relationships between green tea and black tea with cancer. However, a recent study explored the impact of white tea on various types of cancer cells, expanding our understanding of its potential ability to combat cancer. The distinct properties of white tea make it promising for the development of novel anticancer treatments 18. Studies have shown that tea can augment the production of antioxidant enzymes, impede the growth of cancer cells, and facilitate apoptosis 19. These findings further highlight the significant role that tea can play in cancer prevention and intervention. However, an extensive investigation of 500,000 Chinese individuals did not find a clear connection between drinking tea and the occurrence of cancer 20. On the other hand, a subsequent study in the U.S. followed 98,786 postmenopausal women aged 50 to 79 years and discovered that those who consumed one or more sugary drinks per day had a greater chance of developing liver cancer and dying from chronic liver illness 21. Undoubtedly, tea has emerged as an exceptional beverage with abundant health benefits. While the potential of white tea for combating cancer cells is a captivating avenue for further research, continued explorations of the intricate relationship between tea consumption and cancer incidence in diverse populations are essential.

An examination of 33,106 teenagers revealed a correlation between a high intake of basic sugars and sweet drinks in youth and a heightened chance of developing typical adenoma, notably rectal adenoma 22. In a separate investigation within the Iowa Women's Health Study group, the consumption of water containing elevated nitrate levels may be associated with the onset of endometrial cancer 23. Likewise, a case-control analysis conducted in Spain explored the potential connection between water consumption levels and prostate cancer risk 24. Approximately 37 prospective cohort studies provided evidence suggesting that consuming added sugars and sugary beverages increases the likelihood of developing cancer 25. The latest prospective study with Swedish adults, totalling 70,832 subjects from the Swedish Mammography Cohort, showed that a high intake of sweetened drinks might increase the chance of biliary tract cancer 26. Findings from a combined analysis of 22 global studies, with a sample of 9,438 cases and 20,451 controls, revealed an inverse connection between tea intake and stomach cancer 27. Recent research has consistently indicated that the consumption of sugar-sweetened beverages is linked to a greater risk of liver cancer 28, as well as pancreatic cancer 29, along with increased mortality from colorectal cancer and an increased risk of early-onset colorectal cancer in women 30. Furthermore, an association between obesity and certain cancer types is known 31.

Both previous and recent studies have shown a correlation between water consumption and the risk of bladder and kidney cancers, primarily due to varying levels of carcinogens present in drinking water 32,33,34. Our findings support the evidence that overall fluid intake is linked to a decrease in bladder cancer risk 35. Specifically, higher water consumption is also associated with a reduced risk of malignancy.

Sex disparities in drinking habits and their impacts on the incidence of malignancies were investigated in our study. We found that different patterns of tea consumption were closely linked to the occurrence of malignant cancers. While most types of tea were associated with an increased risk of malignancy, white tea was an exception. Regular consumption of white tea significantly decreased the likelihood of developing malignant tumours, particularly in males. However, tea consumption did not affect the incidence of malignancy in females. Interestingly, the source of drinking water did not have any significant impact on the risk of malignancy, except for a possible preventive effect of moderate water intake (less than 2100 ml). This effect was more pronounced in males consuming 1500-1699 ml of water per day. In contrast, our study revealed no association between coffee consumption and the risk of malignancy in either men or women. However, the amount of sugar added to coffee was positively correlated with the risk of developing malignancy, with higher sugar intake leading to a greater risk. This trend was consistent across both sexes. Surprisingly, our findings revealed a reverse association between sugary beverage consumption and the malignancy risk. The more sugary beverages consumed, the lower the risk of developing malignancy, and this effect was observed in both men and women.

Overall, our study highlights the complex relationships between drinking habits and the incidence of malignancies. While white tea, moderate water, and sugary beverage intake may have protective effects, caution should be exercised when adding sugar to coffee. Further research is needed to better understand these patterns and their implications for cancer prevention.

Our study has several limitations. First, the potential for inaccuracies in categorizing caffeine, coffee, and tea intake is a notable limitation because we solely relied on baseline intake information, which may not have accurately reflected participants' actual consumption patterns over time. Moreover, the lack of follow-up data on the consumption of different types of tea is another limitation of our study. By not distinguishing between various types of tea, we may have overlooked potential differences in their cancer risk profiles.

Additionally, data on additional possible cancer risk factors, including familial history, exposure to radiation or chemicals, and dietary patterns, were not available in our study. These factors could have confounded our findings or influenced the observed associations. Consequently, the applicability of our findings to a wider population could be restricted.

In summary, our findings suggest a sex difference in the cancer risk for men and women who consume sweetened coffee and beverages. Specifically, according to our research, consuming large amounts of sweetened caffeinated beverages might lower the likelihood of cancer among males. Nevertheless, these results should be interpreted carefully in light of the mentioned constraints. Moving forward, we hope that our study will promote further research aimed at identifying the specific components of coffee and tea responsible for this potential protective effect. Additionally, exploring the role of sugar in cancer risk would also be of interest.

## Figures and Tables

**Table 1:** Participants' baseline characteristics categorized based on malignancy occurrence

Variable	All participants (n=21916)	No incident malignant tumours (n=21797)	Incident malignant tumours (n=119)	*P* value
Sex, n (%)				0.408
Male	10958 (50.0)	10903 (50.0)	55 (46.2)	
Female	10958 (50.0)	10894 (50.0)	64 (53.8)	
Age, n (%)				0.184
12-17 years	2072 (9.5)	2063 (9.5)	9 (7.6)	
18-59 years	15647 (71.4)	15588 (71.5)	59 (49.6)	
≥60 years	4197 (19.2)	4146 (19.0)	51 (42.9)	
Ethnic groups, n (%)				0.407
Han	19970 (91.1)	19859 (91.1)	8 (6.7)	
Minority	1946 (8.9)	1938 (8.9)	111 (93.3)	
BMI, Mean±SD	21.55±4.12	21.55±4.12	21.98±3.72	
Smoking, n (%)				0.013
No	17901 (81.7)	17816 (81.7)	85 (71.4)	
Ordinary cigarettes	2731 (12.5)	2708 (12.4)	23 (19.3)	
Electronic cigarettes	248 (1.1)	248 (1.1)	0	
Ordinary and electronic cigarettes	279 (1.3)	276 (1.3)	3 (2.5)	
Quit smoking	757 (3.5)	749 (3.4)	8 (6.7)	
Wine consumption, n (%)				0.234
0-25 ml	19607 (89.5)	19504 (89.5)	103 (86.6)	
26-50 ml	1010 (4.6)	1000 (4.6)	10 (8.4)	
51-150 ml	720 (3.3)	717 (3.3)	3 (2.5)	
151-450 ml	381 (1.7)	378 (1.7)	3 (2.5)	
>451 ml	198 (0.9)	198 (0.9)	0	
Tea consumption, n (%)				<0.001
No	10911 (49.8)	10872 (49.9)	39 (32.8)	
Green tea	411 (1.9)	407 (1.9)	4 (3.4)	
Black tea	245 (1.1)	241 (1.1)	4 (3.4)	
Oolong tea	2165 (9.9)	2151 (9.9)	14 (11.8)	
White tea	1670 (7.6)	1665 (7.6)	5 (4.2)	
Yellow tea	118 (0.5)	115 (0.5)	3 (2.5)	
Dark green tea	173 (0.8)	170 (0.8)	3 (2.5)	
Compressed tea	4526 (20.7)	4491 (20.6)	35 (29.4)	
Scented tea	979 (4.5)	971 (4.5)	8 (6.7)	
Others	718 (3.3)	714 (3.3)	4 (3.4)	
Drinking water, n (%)				0.017
≤1200 ml	6351 (29.0)	6319 (29.0)	32 (26.9)	
1200-1499 ml	8037 (36.7)	7994 (36.7)	43 (36.1)	
1500-1699 ml	4805 (21.9)	4784 (21.9)	21 (17.6)	
1700-2099 ml	1621 (7.4)	1612 (7.4)	9 (7.6)	
≥2100 ml	1102 (5.0)	1088 (5.0)	14 (11.8)	
Source of drinking water, n (%)				0.016
Running water	11221 (51.2)	11165 (51.2)	56 (47.1)	
Plain boiled water	2526 (11.5)	2513 (11.5)	13 (10.9)	
Bottled water	1779 (8.1)	1773 (8.1)	6 (5.0)	
Well water	3349 (15.3)	3335 (15.3)	14 (11.8)	
Barrelled water	863 (3.9)	857 (3.9)	6 (5.0)	
Home water purifier	357 (1.9)	352 (1.6)	5 (4.2)	
Community water purifier	1769 (8.1)	1751 (8.0)	18 (15.1)	
Others	52 (0.2)	51 (0.2)	1 (0.8)	
Coffee consumption classification, n (%)				<0.001
None	16994 (77.5)	16919 (77.6)	75 (63.0)	
Decaf coffee	527 (2.4)	522 (2.4)	5 (4.2)	
Instant coffee	211 (1.0)	209 (1.0)	2 (1.7)	
Abrasive coffee	409 (1.9)	406 (1.9)	3 (2.5)	
other coffee	2250 (10.3)	2238 (10.3)	12 (10.1)	
Don't know	1525 (7.0)	1503 (6.9)	22 (18.5)	
Coffee consumption per day, n (%)				0.032
None	16994 (77.5)	16889 (77.5)	105 (88.2)	
<250 ml	2715 (12.4)	2711 (12.4)	4 (3.4)	
250 ml	1140 (5.2)	1135 (5.2)	5 (4.2)	
500 ml	583 (2.7)	582 (2.7)	1 (0.8)	
750 ml	152 (0.7)	151 (0.7)	1 (0.8)	
>750 ml	332 (1.5)	329 (1.5)	3 (2.5)	
Amount of sugar in coffee, n (%)				<0.001
No	19594 (89.1)	19438 (89.2)	86 (72.3)	
<2.5 g	858 (3.9)	852 (3.9)	6 (5.0)	
2.5 g	687 (3.1)	680 (3.1)	7 (5.9)	
5 g	584 (2.7)	575 (2.6)	9 (7.6)	
10 g	173 (0.8)	168 (0.8)	5 (4.2)	
15 g	90 (0.4)	84 (0.4)	6 (5.0)	
Sugary beverages per week				<0.001
Never	8403 (38.3)	8331 (38.2)	72 (60.5)	
<900 ml	9565 (43.8)	9557 (43.8)	38 (31.9)	
1200-1800 ml	2768 (12.6)	2761 (12.7)	7 (5.9)	
>2100 ml	1150 (5.2)	1148 (5.3)	2 (1.7)	
Strenuous exercise per week, n (%)				0.266
None	12599 (57.5)	12534 (57.5)	65 (54.6)	
1 day	2443 (11.1)	2431 (11.2)	12 (10.1)	
2 days	2339 (10.7)	2330 (10.7)	9 (7.6)	
3 days	1496 (6.8)	1483 (6.8)	13 (10.9)	
4 days	1074 (4.9)	1069 (4.9)	5 (4.2)	
5 days	618 (2.8)	613 (2.8)	5 (4.2)	
6 days	406 (1.9)	401 (1.8)	5 (4.2)	
7 days	941 (4.3)	936 (4.3)	5 (4.2)	
The mean time of strenuous exercise per day ± SD (minutes)	25.97±40.30	25.98±40.29	23.71±42.50	0.696
Moderate physical activities per week, n (%)				0.029
None	9953 (45.4)	9899 (45.4)	54 (45.4)	
1 day	2990 (13.6)	2982 (13.7)	8 (6.7)	
2 days	2564 (11.7)	2548 (11.7)	16 (13.4)	
3 days	2466 (11.3)	2446 (11.2)	20 (16.8)	
4 days	1309 (6.0)	1298 (6.0)	11 (9.2)	
5 days	956 (4.4)	950 (4.4)	6 (5.0)	
6 days	392 (1.8)	389 (1.8)	3 (2.5)	
7 days	1286 (5.9)	1285 (5.9)	1 (0.8)	
Mean time of moderate physical activities per day (minutes)	105.83±180.04	105.60±179.60	148.77±245.57	<0.001
Mean amount of walking per day (minutes)	5.01±2.13	5.02±2.31	4.18±2.59	0.007
Mean time of sitting per day (minutes)	447.23±293.58	447.21±293.71	450.99±269.73	0.347

**Table 2:** The relationship between various beverage types and the malignancy risk among participants in the Chinese Health Study (N = 21,916)

Beverage	Cases	Persons	#Model 1 OR (95% CI)	*Model 2 OR (95% CI)	&Model 3 OR (95% CI)
Tea consumption n					
No	39	10911	1.000	1.000	1.000
Green tea	4	411	2.987(1.059-8.425)	2.398(0.837-6.868)	1.894(0.638-5.620)
Black tea	4	245	4.311(1.520-12.226)	4.180(1.456-12.001)	3.024(0.934-9.786)
Oolong tea	14	2165	1.867(1.010-3.451)	1.676(0.900-3.120)	1.536(0.807-2.925)
White tea	5	1670	0.980(0.385-2.499)	0.968(0.378-2.480)	0.815(0.310-2.144)
Yellow tea	3	118	6.215(1.881-20.532)	4.113(1.191-14.202)	4.033(1.102-14.765)
Dark green tea	3	173	5.490(1.646-18.312)	4.206(1.201-14.728)	3.754(0.991-14.220)
Compressed tea	35	4526	2.243(1.412-3.563)	2.199(1.379-3.508)	2.023(1.250-3.273)
Scented tea	8	979	2.546(1.184-5.476)	2.311(1.067-5.005)	1.933(0.866-4.315)
Others	4	718	1.832(0.651-5.160)	1.494(0.522-4.272)	1.232(0.417-3.643)
*P* for trend			0.001	0.008	0.064
Source of drinking water					
Running water	56	11221	1.000	1.000	1.000
Plain boiled water	13	2536	1.119(0.610-2.052)	1.140(0.620-2.097)	0.916(0.487-1.722)
Bottled water	6	1779	0.879(0.375-2.063)	0.812(0.345-1.910)	0.592(0.244-1.438)
Well water	14	3349	1.017(0.562-1.839)	1.049(0.579-1.902)	0.945(0.517-1.728)
Barrelled water	6	863	1.659(0.710-3.876)	1.595(0.679-3.749)	1.593(0.688-3.795)
Home water purifier	5	357	2.518(0.996-6.364)	1.997(0.774-5.152)	1.286(0.452-3.662)
Community water purifier	18	1769	2.134(1.249-3.649)	1.831(1.062-3.160)	0.815(0.357-1.860)
Others	1	52	4.749(0.640-35.237)	2.762(0.300-25.433)	1.623(0.765-3.422)
*P* for trend			0.055	0.323	0.298
Coffee consumption classification					
None	75	16994	1.000	1.000	1.000
Decaf coffee	5	527	3.051(1.217-7.650)	2.551(1.003-6.492)	1.340(0.464-3.865)
Instant coffee	2	211	2.937(0.710-12.138)	2.210(0.521-9.372)	0.497(0.084-2.926)
Abrasive coffee	3	409	2.215(0.691-7.100)	1.726(0.531-5.609)	0.575(0.144-2.305)
Other coffee	12	2250	1.688(0.906-3.147)	1.543(0.821-2.901)	0.815(0.357-1.860)
Don't know	22	1525	4.436(2.716-7.245)	3.608(2.165-6.013)	1.623(0.765-2.422)
*P* for trend			<0.001	<0.001	0.295
Drinking water					
≤1200 ml	32	6351	1.000	1.000	1.000
1200-1499 ml	43	8037	0.941(0.593-1.493)	0.971(0.609-1.548)	0.963(0.597-1.553)
1500-1699 ml	21	4805	0.733(0.420-1.279)	0.724(0.411-1.274)	0.689(0.385-1.233)
1700-2099 ml	9	1621	0.963(0.457-2.030)	0.925(0.433-1.974)	0.756(0.340-1.682)
≥2100 ml	14	1102	2.256(1.354-4.825)	2.692(1.402-5.171)	2.253(1.130-4.493)
*P* for trend			0.007	0.005	0.027
Coffee consumption per day					
None	86	16994	1.000	1.000	1.000
<250 ml	6	2715	0.246(0.091-0.669)	0.251(0.092-0.685)	0.339(0.122-0.942)
250 ml	7	1140	0.739(0.300-1.818)	0.760(0.306-1.885)	0.980(0.387-2.481)
500 ml	9	583	0.288(0.040-2.067)	0.260(0.036-1.891)	0.344(0.047-2.531)
750 ml	5	152	1.008(0.139-7.286)	0.970(0.132-7.104)	1.095(0.041-8.532)
>750 ml	6	332	1.381(0.435-4.385)	1.387(0.425-4.524)	1.279(0.372-4.397)
*P* for trend			0.085	0.089	0.352
Amount of sugar in coffee					
None	86	19524	1.000	1.000	1.000
<2.5 g	6	858	1.926(0.836-4.438)	1.633(0.699-3.811)	1.269(0.452-3.563)
2.5 g	7	687	2.968(1.359-6.437)	2.216(0.995-4.938)	1.764(0.645-4.824)
5 g	9	584	4.780(2.367-9.650)	3.850(1.876-7.902)	3.518(1.406-8.805)
10 g	5	173	7.968(3.145-19.883)	6.316(2.452-16.272)	5.690(1.816-17.830)
15 g	6	90	18.433(7.730-43.955)	10.685(4.180-27.312)	9.362(2.680-32.709)
*P* for trend			<0.001	<0.001	0.001
Sugary beverages per week					
Never	72	8403	1.000	1.000	1.000
<900 ml	38	9595	0.474(0.320-0.704)	0.476(0.319-0.710)	0.553(0.366-0.835)
1200-1800 ml	7	2768	0.295(0.135-0.642)	0.294(0.134-0.646)	0.365(0.164-0.812)
>2100 ml	2	1150	0.199(0.049-0.814)	0.188(0.046-0.774)	0.219(0.052-0.917)
*P* for trend			<0.001	<0.001	0.002

CI, confidence interval; OR, odds ratio. #Model 1 was adjusted for age at recruitment, sex, drinking habit groups, nationality, and BMI. *Model 2 was modified to include smoking, alcohol consumption, weekly strenuous exercise, daily strenuous exercise, weekly moderate physical activity, daily moderate physical activity, daily walking, and daily sitting, in addition to the variables in Model 1. &Model 3 was adjusted for all variables in Model 2, plus the intake of different types of tea, source of drinking water, coffee consumption classification, volume of drinking water per day, coffee consumption per day, amount of sugar in coffee, and sugary beverages per week.

**Table 3:** Sex differences in tea consumption and the risk of malignancy

Tea consumption	No	Green tea	Black tea	Oolong tea	White tea	Yellow tea	Dark green tea	Compressed tea	Scented tea	Others	*P* for trend
Male											
#Model 1 OR (95% CI)	1.000	1.589 (0.209-12.709)	6.188 (1.770-21.627)	2.500 (1.101-5.681)	0.618 (0.082-4.676)	13.497 (3.813-47.781)	11.929 (3.341-42.589)	2.868 (1.506-5.464)	2.117 (0.614-7.308)	2.181 (0.497-9.563)	<0.001
*Model 2 OR (95% CI)	1.000	1.079 (0.137-8.492)	5.948 (1.668-21.205)	2.287 (0.990-5.283)	0.718 (0.094-5.478)	8.173 (2.131-31.348)	8.257 (2.077-32.823)	2.785 (1.448-5.358)	1.757 (0.497-6.212)	1.813 (0.403-8.165)	0.005
&Model 3 OR (95% CI)	1.000	1.023 (0.125-8.388)	5.563 (1.479-20.925)	2.016 (0.829-4.902)	0.736 (0.095-5.704)	7.350 (1.739-31.063)	5.453 (1.04-27.192)	2.680 (1.358-5.286)	1.370 (0.361-5.200)	1.464 (0.305-7.040)	0.033
Female											
#Model 1 OR ( 95% CI)	1.000	4.537 (1.341-15.351)	2.496 (0.332-18.970)	1.335 (0.505-3.526)	1.118 (0.383-3.565)	0	0	1.736 (0.860-3.535)	3.135 (1.170-8.403)	1.661 (0.387-7.129)	0.292
*Model 2 OR (95% CI)	1.000	3.770 (1.065-13.342)	1.811 (0.204-16.100)	1.170 (0.437-3.134)	1.065 (0.361-3.141)	0	0	1.639 (0.805-3.338)	2.858 (1.054-7.752)	1.202 (0.268-5.378)	0.506
&Model 3 OR (95% CI)	1.000	3.334 (0.892-12.532)	0.811 (0.029-22.359)	1.104 (0.399-3.056)	0.902 (0.283-2.873)	0	0	1.608 (0.767-3.371)	2.909 (1.028-8.232)	1.393 (0.308-6.292)	0.579

**Table 4:** Sex differences in the source of drinking water and the risk of malignancy

Source of drinking water	Running water	Plain boiled water	Bottled water	Well water	Barrelled water	Home water purifier	Community water purifier	Others	*P* for trend
Male									
#Model 1 OR (95% CI)	1.000	1.409 (0.637-3.114)	0.501 (0.118-2.129)	1.162 (0.523-2.582)	1.805 (0.543-6.008)	3.063 (0.913-10.272)	2.549 (1.284-5.060)	9.925 (1.272-77.467)	0.033
*Model 2 OR (95% CI)	1.000	1.472 (0.660-3.282)	0.456 (0.107-1.951)	1.151 (0.514-2.577)	1.474 (0.433-5.020)	2.168 (0.607-7.737)	2.255 (1.113-4.571)	5.300 (0.462-60.854)	0.219
&Model 3 OR (95% CI)	1.000	0.970 (0.402-2.340)	0.309 (0.065-1.473)	0.933 (0.406-2.144)	1.193 (0.422-4.427)	1.531 (0.385-6.086)	2.448 (1.179-5.081)	2.102 (0.088-50.363)	0.201
Female									
#Model 1 OR (95% CI)	1.000	0.841 (0.324-2.181)	1.483 (0.511-4.306)	0.867 (0.356-2.109)	1.514 (0.457-5.017)	1.984 (0.463-8.493)	1.665 (0.687-4.037)	0	0.856
*Model 2 OR (95% CI)	1.000	0.869 (0.333-2.565)	1.445 (0.494-4.266)	0.921 (0.376-2.257)	1.639 (0.490-5.482)	1.784 (0.391-8.137)	1.435 (0.584-3.530)	0	0.942
&Model 3 OR (95% CI)	1.000	0.804 (0.300-2.153)	1.091 (0.354-3.357)	0.773 (0.308-1.940)	1.752 (0.512-5.992)	1.677 (0.282-9.967)	1.325 (0.511-3.434)	0	0.949

**Table 5:** Sex differences in the coffee consumption classification and risk of malignancy

Coffee consumption classification	None	Decaf coffee	Instant coffee	Abrasive Coffee	Other coffee	Don't know	*P* for trend
Male							
#Model 1 OR (95% CI)	1.000	2.517 (0.596-10.626)	4.741 (1.118-20.093)	3.908 (0.926-16.490)	2.462 (1.131-5.357)	4.398 (2.212-8.746)	<0.001
*Model 2 OR (95% CI)	1.000	2.067 (0.478-8.948)	3.430 (0.759-15.497)	3.041 (0.695-13.312)	2.197 (0.984-4.906)	3.252 (1.560-6.778)	0.021
&Model 3 OR (95% CI)	1.000	1.127 (0.222-5.729)	0.856 (0.115-6.367)	0.975 (0.155-6.130)	1.309 (0.438-3.913)	1.478 (0.500-4.370)	0.974
Female							
#Model 1 OR (95% CI)	1.000	3.602 (1.086-11.945)	0	1.113 (0.151-8.212)	1.008 (0.352-2.889)	4.346 (2.153-8.773)	0.001
*Model 2 OR (95% CI)	1.000	3.004 (0.883-10.225)	0	0.977 (0.131-7.292)	0.944 (0.325-2.743)	3.780 (1.830-7.807)	0.009
&Model 3 OR (95% CI)	1.000	1.464 (0.336-6.376)	0	0.346 (0.035-3.453)	0.475 (0.123-1.841)	1.654 (0.558-4.903)	0.336

**Table 6:** Sex differences in the volume of drinking water and risk of malignancy

Drinking water	≤1200 ml	1200-1499 ml	1500-1699 ml	1700-2099 ml	≥2100 ml	*P* for trend
Male						
#Model 1 OR (95% CI)	1.000	1.254 (0.642-2.449)	0.618 (0.263-1.451)	1.424 (0.565-3.589)	3.048 (1.291-7.197)	0.017
*Model 2 OR (95% CI)	1.000	1.311 (0.663-2.595)	0.611 (0.256-1.460)	1.441 (0.560-3.711)	3.146 (1.289-7.676)	0.016
&Model 3 OR (95% CI)	1.000	1.290 (0.632-2.634)	0.545 (0.220-1.348)	1.172 (0.422-3.250)	2.989 (1.150-7.770)	0.022
Female						
#Model 1 OR (95% CI)	1.000	0.692 (0.357-1.341)	0.905 (0.435-1.886)	0.483 (0.112-2.087)	2.128 (0.788-5.744)	0.219
*Model 2 OR (95% CI)	1.000	0.718 (0.368-1.400)	0.900 (0.426-1.903)	0.466 (0.105-2.075)	2.302 (0.825-6.423)	0.213
&Model 3 OR (95% CI)	1.000	0.736 (0.369-1.467)	0.892 (0.408-1.949)	0.425 (0.091-1.997)	2.174 (0.725-6.516)	0.300

**Table 7:** Sex differences in the amount of sugar added to the coffee and the risk of malignancy

Amount of sugar added to coffee	None	<2.5 g	2.5 g	5 g	10 g	15 g	*P* for trend
Male							
#Model 1 OR (95% CI)	1.000	2.464 (0.878-6.920)	4.213 (1.643-10.799)	6.149 (2.375-15.919)	2.975 (0.402-22.006)	20.938 (7.093-61.808)	<0.001
*Model 2 OR (95% CI)	1.000	2.121 (0.731-6.154)	2.546 (0.932-6.957)	5.192 (1.922-14.030)	2.626 (0.340-20.303)	11.807 (3.593-38.802)	<0.001
&Model 3 OR (95% CI)	1.000	1.403 (0.352-5.586)	1.588 (0.408-6.181)	4.096 (1.115-15.049)	1.750 (0.183-16.778)	11.243 (2.224-56.843)	0.049
Female							
#Model 1 OR (95% CI)	1.000	1.316 (0.315-5.494)	1.681 (0.402-7.036)	3.694 (1.299-10.505)	12.986 (4.477-37.662)	14.881 (3.405-65.031)	<0.001
*Model 2 OR (95% CI)	1.000	1.068 (0.247-4.610)	1.410 (0.332-5.985)	3.184 (1.099-9.220)	12.067 (3.978-36.598)	8.273 (1.535-44.583)	<0.001
&Model 3 OR (95% CI)	1.000	1.294 (0.240-6.974)	1.264 (0.229-6.978)	3.341 (0.852-13.103)	15.870 (3.663-68.759)	5.896 (0.594-58.536)	0.005

**Table 8:** Sex differences in sugars consumed per week and the risk of malignancy

Sugary beverages per week	Never	<900 ml	1200-1800 ml	≥2100 ml	*P* for trend
Male					
Model 1 OR (95% CI)	1.000	0.377 (0.214-0.666)	0.375 (0.148-0.952)	0.185 (0.025-1.350)	0.002
Model 2 OR (95% CI)	1.000	0.392 (0.22-0.698)	0.394 (0.153-1.017)	0.175 (0.024-1.291)	0.003
Model 3 OR (95% CI)	1.000	0.461 (0.251-0.847)	0.492 (0.183-1.323)	0.179 (0.023-1.399)	0.031
Female					
Model 1 OR (95% CI)	1.000	0.612 (0.35-1.069)	0.197 (0.047-0.827)	0.223 (0.03-1.641)	0.039
Model 2 OR (95% CI)	1.000	0.599 (0.34-1.055)	0.187 (0.044-0.793)	0.173 (0.023-1.321)	0.027
Model 3 OR (95% CI)	1.000	0.665 (0.369-1.198)	0.24 (0.056-1.031)	0.206 (0.026-1.623)	0.096

## Abbreviations

OR: Odds ratio
CIs: Confidence intervals
BMI: Boss mass index
SD: Standard deviation
